# Medical Miscellany

**Published:** 1840-07

**Authors:** 


### MEDICAL MISCELLANY.

Dr. Thomas W. Colescott, of Cincinnati, is engaged in translating the admirable work of *Dr. Civiale*, on *Calculous Affections*, recently published in Paris. The work will be ready for the press early in the winter, and from the ability of Dr. C. there is every reason to believe that the translation will be well executed. It will be comprised in an 8vo. volume of about 500 pages.

*Medical College of St. Louis.*—A medical department has been attached to the Kemper College, of St. Louis, under the charter of that institution. The Faculty has been organized, and consists of the following members: Joseph N. McDowell, M.D., Professor of Anatomy and Surgery; J. W. Hall, M.D., (late of this city,) Professor of the Theory and Practice of Medicine. Hiram M. Prout, M.D., Professor of Materia Medica and Medical Botany. John S. Moore, M.D., Professor of the Institutes of Medicine and Obstetrics. John De Wolf, M.D., Professor of Chemistry and Pharmacy.

The work of Professor Gross, on Pathological Anatomy, has received the commendation of all the leading Journals of the country—a Review of it has been prepared for our next number.

We have received, from Professor Beck, the Transactions of the Medical Society of the State of New York, for 1839 and 1840, which we shall notice in an early number. Y.

